# Multiple use applicator for vaginal tablets/vaginal inserts: compliance verification and suitability studies

**DOI:** 10.1186/s12905-020-01099-y

**Published:** 2020-10-15

**Authors:** Heiko Brunner, Rudolf A. Theodor

**Affiliations:** HELM AG, Nordkanalstrasse 28, 20097 Hamburg, Germany

**Keywords:** Estradiol, Vaginal applicator, Multiple use, Vaginal tablet, Vaginal insert, Generic, Vagifem®, Cleaning verification, Simulated artificial vaginal fluid, Mechanical stability, Plastic waste reduction

## Abstract

**Background:**

A newly developed multiple use applicator for vaginal tablets/vaginal inserts* was evaluated for its general suitability. There are no standard procedures described in guidelines or general accepted publications as to how this kind of product should be tested for suitability of purpose.

**Methods:**

Due to the lack of existing standard procedures, three separate tests were designed and successful executed: (a) First, a patient acceptability evaluation was carried out as part of a phase III trial (registered in EudraCT on 9 Jan 2017, number 2017-000142-22 2. https://www.clinicaltrialsregister.eu/ctr-search/search?query=eudract_number%3A2017-000142-22). (b) Secondly, a cleaning procedure for the applicator after simulated multiple use was developed and verified in order to prove a minimized risk of microbiological contamination of the device. A newly developed vaginal fluid to simulate multiple applications was applied for this trial. (c) Lastly, a third trial evaluated the mechanical stability and proper functionality of the applicator after multiple simulated uses. Even potential abrasion of material of the device was checked.

**Results:**

Acceptable patient compliance of the new multiple use applicator was verified after 2 weeks of daily use. Furthermore, diary data assessments of patients participating in the Pharmacodynamic part of the trial were evaluated as well. Overall, patient acceptability of the new applicator was proven. The easy-to-use cleaning procedure for the applicator, which can even be carried out in a domestic environment, was developed and successfully verified for effectiveness, meeting all microbiological acceptance criteria for vaginal products of the European Pharmacopeia. The mechanical stability and proper functionality of the applicator after 50 simulated uses was also evaluated. All tested applicator batches (fresh and aged) passed the final evaluation, with no limitations in functionality.

**Conclusion:**

The tests developed and executed consider various compliance aspects of the newly developed applicator. Results of these individual tests met the expectations and/or the predefined acceptance criteria. All included trials performed produced results justifying and qualifying the applicator for the intended multiple-use. The procedures outlined may also be a guide as to how this kind of medical device can be tested for suitability.

*Trial registration* Registered in EudraCT, number 2017-000142-22, start date 24 May 2017.

## Background

A clinical phase III study to assess the therapeutical equivalence of a newly developed vaginal tablet containing 10 µg of estradiol in comparison with a marketed originator reference product (Vagifem®) was performed [1]. The indication is treatment of vaginal atrophy in postmenopausal women.

In line with the site of administration, close to the site of action, estradiol-containing vaginal tablets are considered as “locally applied, locally acting” drug products. Applicators were used for the application of the medicinal product, to insert the tablet to the location of action.

The treatment scheme consisted of daily applications of one tablet for two weeks followed by the application of one tablet twice a week for up to 5 weeks. This results in up to 7 weeks of therapy, with the application of up to 24 tablets.

The originator product consists of a single use, pre-filled applicator containing one tablet.

The newly developed product consists of tablets in a blister (14 or 24 tablets) and a separate applicator (class I medical device) for multiple use. The patient has to remove a tablet from the blister and insert the tablet into the applicator prior to application of the tablet to the intra-vaginal site of action.

The concept of a multiple-use applicator vs. a single-use applicator was chosen to reduce both production and waste of plastic material, decreased amount of packaging material due to overall smaller pack sizes and to minimize transportation and storage related expenses.

Although the device is only a class I medical device, several trials were performed as the application of vaginal products is a sensitive topic. Patient compliance is a key factor in the success of treatment.

Therefore, the performed trials for compliance verification and suitability testing of the applicator cover the entire range from handling, cleaning and mechanical stability/strength of the applicator.

Three tests were performed to qualify the newly designed applicator for human use and for the multiple use application. Those three tests include:

- Usability of the Applicator Handling by Patients (including complaint monitoring during CT Phase III)
- Cleaning Verification Trial
- Mechanical Functionality and Stability Trial

The first test focused on the applicator handling by the patients and the usability of the applicator for the patients.

Tests 2 and 3 qualified the applicator for multiple use.

The design of the tests considered the requirements of the Medical Device Directive 93/42/EEC and of the Medical Device Regulation (EU) 2017/745, respectively.

## Methods

1. Usability trial

**Fig. 1:**
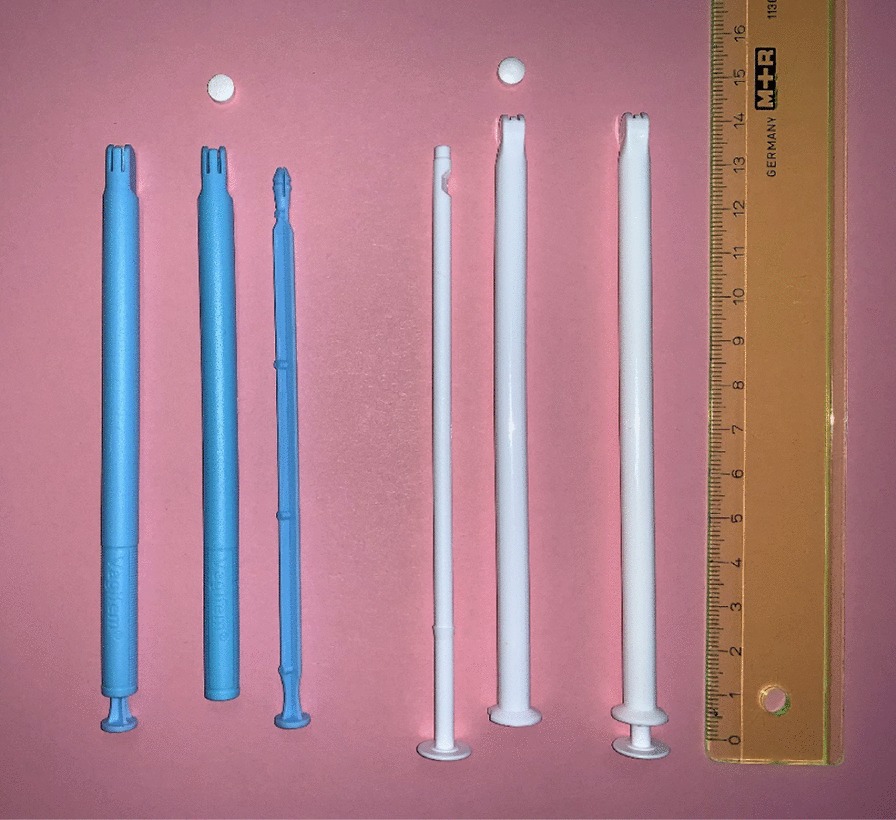
Originator product (left) and newly developed product (right), both applicators consisting of a tube and a plunger

As the applicator of the newly developed product was designed to be very comparable to the applicator of the originator product (see Fig. 1), a separate intensive trial, comparable to a human factor study [2], matching patient needs as required for US bound medical devices has not been performed for the new applicator.

Both applicators have a very comparable size and consist of a tube and a plunger. General functionality of both devices like tablet holding and release of the tablet is identical.

Patients participating in the assessment of the applicator usability, used only the HELM product "Estradiol 10 µg vaginal tablets and applicator" during the characterisation of systemic exposure with estradiol over a period of 14 days of once daily application.

In total, 13 subjects included in the HELM product "Estradiol 10 µg vaginal tablets and applicator" treatment group of the pharmacokinetic arm of the clinical trial were asked to complete a questionnaire evaluating the handling properties of the newly developed applicator at the end of the 2 weeks trial period. The questionnaire was specifically developed for the study. A translation of the questionnaire is provided hereafter.

### Questionnaire


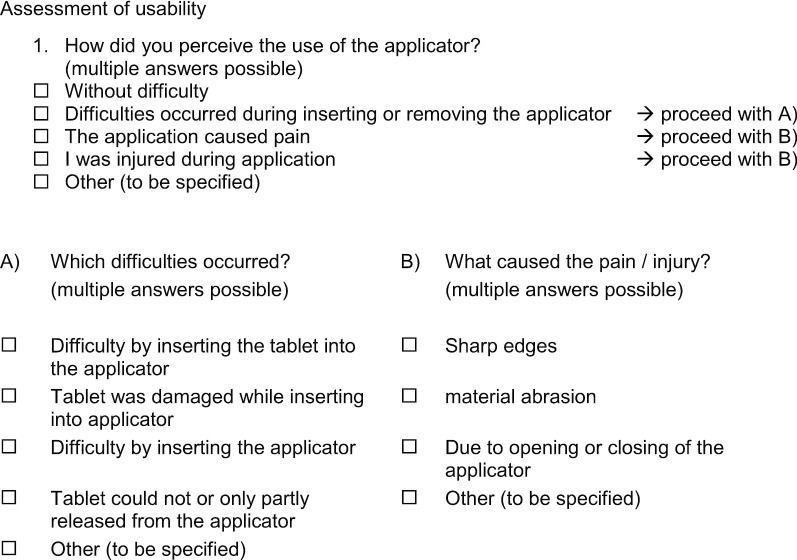
Since the questionnaire about applicator handling was completed by the patients only upon the conclusion of the 2 weeks trial period, patients rated all 14 occasions of applicator use (vaginal tablets were applied once daily for 14 days). Only 1 out of 13 subjects reported “some difficulties during insertion and removal,” with the remaining 12 subjects reporting the applicator handling experience as “problem free”. Therefore, with 12 subjects rating the applicator handling as “problem free” at all 14 occasions of use for vaginal application of the tablet, this adds up to 168 problem free usages of the HELM product "Estradiol 10 µg vaginal tablets and applicator" out of a possible total of 182 applications (Table 1).

**Table 1:** Summary of user compliance study

Patients (description)	N	Percentage (%)
Patients participating in trial part	13	100
Number of applications	182	100
Number of applications w/o Problems	168	92.3

Only 1 patient reported having problems, though these problems were a result of general aversion to the concept of applicator use by the patient. This patient described "difficulty in managing the applicator without prematurely pressing the release mechanism prior to placing the tablet correctly", "feeling of uneasiness when holding the applicator while placing it properly", "feeling of unhygienic manipulation", and the replies were based on a general non-acceptance of the applicator principle. The problems described by the patient are independent of the product and would also perfectly describe the procedure for the Vagifem® applicator.

Patients participated in this trial was all post-menopausal. Ages ranged from 49 to 74 years with a mean age of 61.5 years and a median of 62 years respectively.

## Results

In summary, it can be concluded that all patients open to the use of a vaginal applicator, did not experience any problems during the usage of the Helm applicator, neither with placing the tablet into the applicator, nor during for the proper placement of the tablet intravaginally using the applicator.

Furthermore, 415 subjects completed the PD part of the trial, using the applicator for a total duration of 6 weeks (2 weeks of daily application, followed by 4 weeks of twice weekly application). Within the subjects’ diary for the PD part of the trial, a section asking for any particularities regarding IMP application had been filled out by each subject on a daily basis.

A total of 9336 applications of vaginal tablets (including cases documented by drop out subjects) were documented for the Helm product "Estradiol 10 µg vaginal tablets and applicator". Analyzing the results of diary data assessments, the following statements can be made:

- 9336 occasions of applicator use occurred in total
- 207 occasions were not reported or entries in diaries were missing
- 9129 occasions of applicator use have been rated by the subjects, which can be separated as follows:
  - 8877 occasions were reported with no particularities concerning usage of the applicator
  - 243 occasions were reported with particularities, but these verifiably did not concern the applicator itself but the timepoint of application or use of reserve tablets
  - 9 occasions were reported with particularities concerning the applicator itself

Therefore, the total number of documented cases where the Helm product "Estradiol 10 µg vaginal tablets and applicator" has been used in the PD part of the clinical trial without reporting problems concerning the applicator handling is 9120 (99.9%) out of 9129 cases reported and assessed by the patients.

Consequently, the vast majority of applications were performed without particularities concerning applicator handling, with applicator handling not being an issue in terms of usability for the patients.

2. Cleaning verification trial

A variety of medicinal vaginal products in the EU are marketed with an applicator for multiple use, including vaginal tablets, gels, creams, ointments, and suppositories. Accordingly, several cleaning methods for multiple use applicators can be found in the internet or within patient information leaflets. The table below (Table 2) gives an overview concerning those products and related cleaning information.

**Table 2:** Marketed vaginal products with multiple-use applicator

No	Product	Cleaning description	Reference/Source
1	Applicator for vaginal gel	“Clean after use”	https://medesign.de/gynaekologie-pessare/abstrichsysteme/gel-applikator-vaginal
2	Applicator for vaginal tablets	“Clean with lukewarm water and mild soap”	https://medesign.de/gynaekologie-pessare/abstrichsysteme/tabletten-applikator-vaginal
3	Applicator for Vaginal creme, Vaginal gel and vaginal tablets, general description HEXAL	Clean with lukewarm water and soap. Rinse applicator with water to remove remaining soap. Let applicator dry by exposure to air prior to next use	https://www.hexal.de/praeparate/arzneimittel-richtig-anwenden/vaginalcreme-vaginaltabletten/
4	Applicator for vaginal tablets	Clean with lukewarm water and mild soap. Rinse applicator with water to remove remaining soap. Let applicator dry by exposure to air prior to next use	https://www.apo-rot.de/details/tabletten-applikator-vaginal/8445718.html
5	Applicator for Vaginal tablets	Clean carefully with warm water (3 days therapy)	https://www.kadefungin.de/wp-content/uploads/gi/kadefungin-3-vaginaltabletten-dr-kade.pdf and
5a			https://beipackzetteln.de/antifungol-hexal-3-vaginaltabletten and
5b			https://studylibde.com/doc/2623954/beipackzettel—fliegende-pillen
6	Applicator for Vaginal suppository, general discription	Clean carefully with water and mild soap	https://de.wikihow.com/Scheidenz%C3%A4pfchen-einf%C3%BChren
7	Applicator for Vaginal tablets	Clean carefully with warm water and soap	https://carefirst.staywellsolutionsonline.com/RelatedItems/121,80594

Even on YouTube, various videos on the use and cleaning of vaginal applicators can be found. In some cases, spare applicators for different types of products are available on the market as well.

In comparison to vaginal gels, creams or ointments, vaginal tablets do not contaminate the applicator much due to their matrix, nevertheless proper cleaning of the applicator after use is a hygiene factor for the patient. An effective and easy to follow cleaning procedure for the home environment was developed. The effectiveness of this cleaning procedure was assessed after simulated multiple use.

The cleaning trial was divided into 4 cleaning verification stages:

- Stage 1: Examination of the microbiological status of an unused applicator
- Stage 2: Examination of the microbiological status of the applicator after 1 simulated use followed by cleaning
- Stage 3: Examination of the microbiological status of the applicator after simulated 14 daily uses, each use followed by cleaning (2 weeks simulated use)
  Stage 4: Examination of the microbiological status of the applicator after simulated 24 uses in accordance with the treatment scheme (1 daily simulated use for 2 weeks (14 uses), followed by simulated twice per week uses for 5 weeks (10 uses), overall test time was 7 weeks), each use of course followed by cleaning.

As a first step, the different cleaning methods were evaluated for acceptable microbiological status after a single simulated use, followed by the application of the corresponding cleaning procedure (Table 3). To simulate use of the applicator, a placebo tablet was placed into the device. The applicator was then dipped for a few seconds into an artificial vaginal fluid containing two different types of lactobacilli and the placebo tablet was released. After removal of the applicator from the artificial vaginal fluid, and an additional one minute waiting interval, the applicator was cleaned following one of four different methods.

**Table 3:** Evaluated cleaning procedures for applicator

Rinsing with	Cold water	Warm water
Using		
No soap	Procedure 1	Procedure 2
Mild soap	Procedure 3	Procedure 4

After cleaning and allowing the applicator air dry, the microbiological status was evaluated. The microbiological acceptance criteria for the cleaning procedure were derived from the European Pharmacopeia specifications for vaginal products [3].

Based on the outcome, the final cleaning method was chosen to be used during multiple simulated use trials (stages 3 and 4).

## Experimental

The cleaning verification trial was performed in three steps. First, the cleaning procedure to be used was evaluated for effectiveness after a simulated single application.

No generally accepted artificial vaginal fluid is described in the literature. The specificity in regard to type and concentration of lactic acid bacteria is very much depent on the individual woman [4, 5], e.g. depending on the status of the Nugent-Score [6] and on the menstrual cycle [7]. However, it is common understanding that the pH value should be within 3.8 to 4.5 for healthy women.

For the documented in-vitro trials simulating in-vivo use, an artificial vaginal fluid containing *Lactobacillus iners* DSM 13,335 and *Lactobacillus crispatus* DSM 20,584 was applied.

The bacterial suspension for the simulated usage was prepared according to the procedures described in Ph. Eur., 9th Edition, using MRS agar (*L. crispatus*) and Columbia Blood agar (*L. iners*) under microaerophilic conditions. The stability of the microorganism was evaluated by daily checks of microbial counts prior to use. A shelf life for the suspension was assigned.

Each bacterial suspension was adjusted to approximately 1 × 10^8^ CFU/mL (CFU: colony forming units) with 0.9% NaCl. The density of the suspensions was confirmed by microbial count determination using the surface spread method (Ph. Eur., 9th Edition) 2.6.12).

For the simulated usage, the two *lactobacilli* suspensions were mixed at a ratio of 1:1 and then diluted in lactic acid buffer pH 4; the microbial density was adjusted to 1 × 10^6^ CFU/mL.

The test description for the vitro studies is:

Procedure for the daily usage simulation:

1. The operator cleaned his/her hands with soap and water
2. For each usage simulation, 5 applicators were each loaded with one placebo tablet
3. 47 mL of the inoculated lactic acid buffer (pH 4) were transferred into a sterile glass tube (15 × 2.5 cm)
4. Each applicator was dipped approximately 10 cm of its length into the inoculated lactic acid buffer for 3 s and the tablet was ejected during this time period
5. After removal of the applicator, it was left to dry for 1 min on a smooth surface without contact between the wet, treated applicator part and the surface
6. Cleaning: The plunger was pulled out of the applicator and both the applicator and plunger were cleaned with one of the following 4 variants of the cleaning procedures (Table 4). The applicator was cleaned inside and out.

**Table 4:** Microbiological results after one simulated use

Option no	Cleaning method	Result after single simulated use
1	Clean with cold water	Out of specification (OOS) TAMC: 1.6 × 10^3^ CFU/Applicator TYMC: 4.17 × 10^2^ CFU/Applicator (Bacteria: 4.15 × 10^2^ CFU/Applicator, molds 2.00 × 10^0^ CFU/Applicator) *S. aureus*: conforms *P. aeruginosa*: conforms *C. albicans*: conforms
2	Clean with warm water	OOS TAMC: 1.04 × 10^2^ CFU/Applicator TYMC: 4.90 × 10^1^ CFU/Applicator *S. aureus*: does not conform *P. aeruginosa*: conforms *C. albicans*: conforms
3	Clean with cold water with soap	TAMC: 7.90 × 10^1^ CFU/Applicator TYMC: 1.00 × 10^1^ CFU/Applicator *S. aureus*: conforms *P. aeruginosa*: conforms *C. albicans*: conforms
4	Clean with warm water with soap	TAMC: 7.00 × 10^0^ CFU/Applicator TYMC: < 2.00 × 10^0^ CFU/Applicator *S. aureus*: conforms *P. aeruginosa*: conforms *C. albicans*: conforms

Tests were performed with n = 5 applicators per option

The microbiological quality of the used applicators was examined according to DIN EN ISO 11737-1 and Ph. Eur., 9th Edition, 2.6.12/2.6.13 with the methods tested for their suitability.

Acceptance criteria for remaining microbiological organisms was derived from the European Pharmacopeia for vaginal products:

TAMC: 4 × 10^2^ CFU/applicator (max. 8 × 10^2^ CFU/applicator).

TYMC: 4 × 10^1^ CFU/applicator (max. 8 × 10^1^ CFU/applicator).

*Pseudomonas aeruginosa*: absent (per applicator).

*Staphylococcus aureus*: absent (per applicator).

*Candida albicans*: absent (per applicator).

Based on the results obtained in the “single use” trial (Table 4), cleaning method No 4 was chosen to be applied in the multi-use study.

In the multiple simulated use trial, the frequency of the application scheme [8] was applied, e.g. daily use for two weeks, followed by twice weekly use for five weeks. This reflects the maximum of 24 applications.

After 14 and 24 simulated usages (Tables 5, 6) the microbiological status was evaluated. For results see Tables 5 and 6.

**Table 5:** Results of microbiological examination of used applicators after 14 simulated usages and application of cleaning procedures

Test parameter	Result	Evaluation
TAMC	66 CFU/applicator	Complies
TYMC	9 CFU/applicator	Complies
Pseudomonas aeruginosa	Absent/applicator	Complies
Staphylococcus aureus	Absent/applicator	Complies
Candida albicans	Absent/applicator	Complies

**Table 6:** Results of microbiological examination of used applicators after 24 simulated usages and application of cleaning procedure

Test parameter	Result	Evaluation
TAMC	2.25 × 10^2^ CFU/applicator	Complies
TYMC	24 CFU/applicator	Complies
Pseudomonas aeruginosa	Absent/applicator	Complies
Staphylococcus aureus	Absent/applicator	Complies
Candida albicans	Absent/applicator	Complies

## Results

The results of the examination of the microbiological quality show that after 14 simulated usages including cleanings with a mild soap and rinsing with warm water over a time period of 14 days, the microbiological quality complies with the requirements of the Ph. Eur. 5.1.4 for products for vaginal use.

Furthermore, the microbiological quality also complies after 24 simulated usages including cleaning with a mild soap and rinsing with warm water over a period of 7 weeks.

Thus, the results indicate that the cleaning procedure applied here is suitable to effectively clean the vaginal applicator for up to 24 multiple usages under the test conditions applied.

3. Mechanical stability and functionality trial

A mechanical stability and functionality trial simulating multiple-use of the applicator was performed. For this trial, placebo tablets were placed into the applicator and ejected up to 50 times per applicator. Parameters evaluated includes:

- Plunger hold while in the lock position
- Tablet hold within the applicator
- Tablet release
- Physical and mechanical intactness of applicator. It was be tested and reported whether segments of the applicator broke, regardless of maintain functionality

Several applicator batches of different ages were tested in this trial (Table 7).

**Table 7:** Used material in the mechanical stability study

Sample group	Applicator^a^ art. no	Batch tube	Batch plunger	Drawing tube	Drawing plunger	Material tube	Material plunger	Age of applicator^a^
1	Tablet applicator “HELM” 1033xx	1650	1649A	16–340	0880	LDPE 780E + 2%PB 8100 white	PP Purell HP570M + 2% PB 8100 white	~ 2 years
2	Tablet applicator “HELM” 1033xx	1709A	1705	16–340	0880	LDPE 780E + 2%PB 8100 white	PP Purell HP570M + 2% PB 8100 white	~ 1 year
3	Tablet applicator “HELM” 1033xx	1716	1713A	16–340	0880	LDPE 780E + 2%PB 8100 white	PP Purell HP570M + 2% PB 8100 white	~ 1 year
4	Tablet applicator 1011xx	1505	1542	04–021	0880	LDPE 780E + 3%PB 8100 white	PP Purell HP570M + 2% PB 8100 white	3 years
5	Tablet applicator 1011xx	1211	1216	04–021	0880	LDPE 780E + 3%PB 8100 white	PP H733-07 + 2% PB 8100 white	6 years

^a^Applicator = unit of assembled tube and plunger

**Fig. 2:**
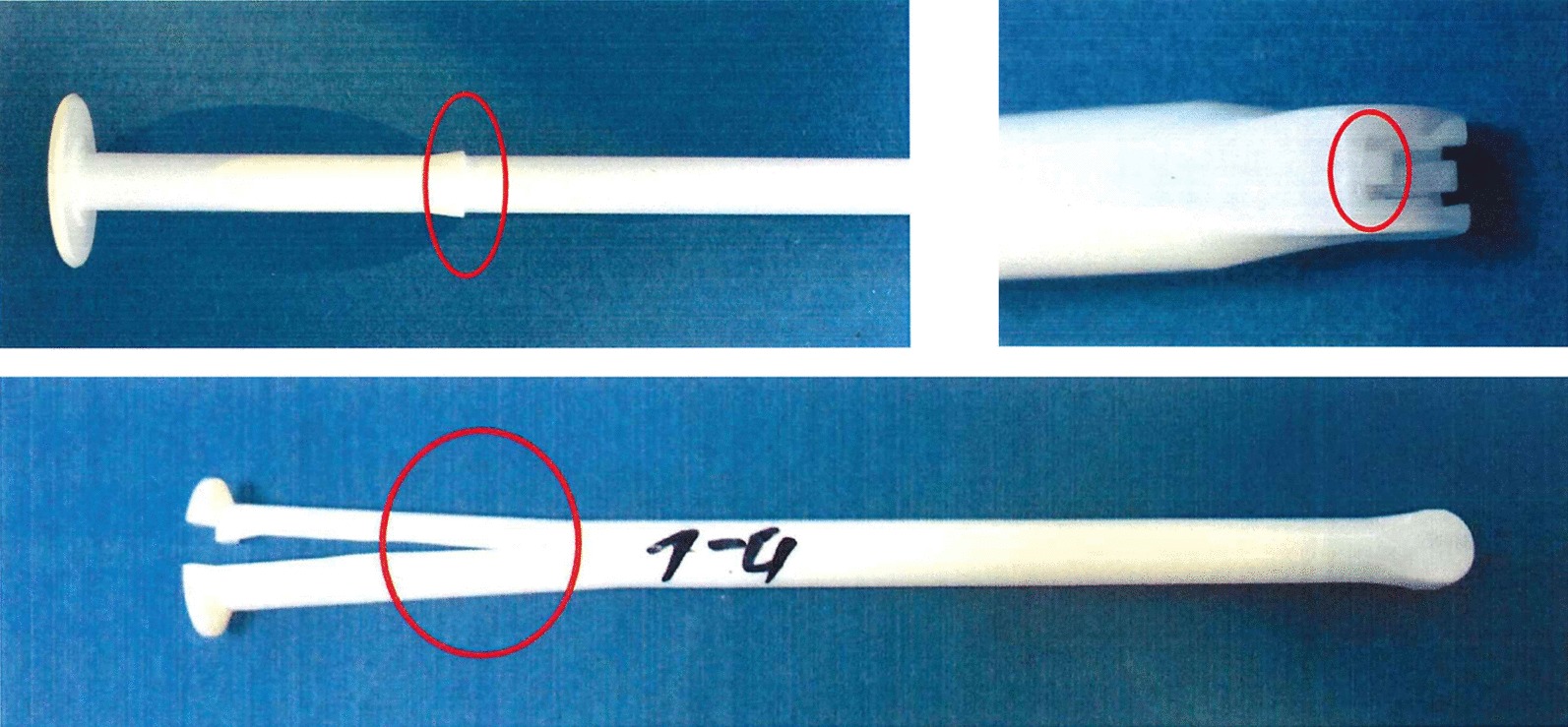
Tube and plunger marked with the critical points for evaluation to ensure proper functionality. Note that the tube (marked with 1–4) was cut after the mechanical stress testing in order to perform the evaluation. No breaking or cracking of the tube was observed during the entire trial

Visual evaluation was performed by stereo microscope (tenfold magnification). The positions of the points of visual inspection of the applicators are shown below (Fig. 2).

## Results

All applicators were fully functional after simulated testing, although some slight mechanical degradation could be observed on some applicator parts after 50 simulated uses. No breaks of any kind were observed.

From these results, the conclusion can be drawn that applicator functionality in terms of

- Plunger hold in lock position
- Tablet hold
- Tablet release

is completely present and sustained after up to 50 cycles of applicator reuse. The trial shows no impact of applicator aging to mechanical stability for at least an applicator age of up to 6 years.

Microscopical results show that with each application the retention ring within the tube is abraded by the plunger retention ring bit by bit. Nevertheless, the functionality in terms of plunger hold is preserved for up to 50 reuse cycles. Microscopical small abrasions and particles can be detected at the retention ring area of the tube after 10 reuse cycles.

The microscopical comparison of the applicators used for the cleaning verification trial showed the same results as the applicators used up to 50 times without intermediate cleaning. For these applicators, the retention ring in the tubes showed visible abrasions as reuse cycles increased, however, with unimpaired usability.

Furthermore, applicators used in the cleaning verification trial (24 simulated uses) were evaluated in accordance with the criteria of this mechanical stability trial and found to be acceptable.

Loose particles from abrasions were not detected in the applicators from the cleaning verification study. It can be concluded that lose particles are eliminated during the cleaning procedure.

## Conclusion

Three different trials covering various aspects of the qualification of a multiple use applicator for vaginal tablets were performed. The aspects include patient compliance, applicator cleaning verification as well as mechanical stability of the applicator covering the timeframe of the intended use.

The applicator for the generic product is very comparable to the applicator of the originator product in terms of design and functionality. A patient trial revealed no issues in the handling of the applicator, neither in regard to placing the tablet into the applicator, nor with the actual use of the applicator for proper placement of the tablet intra-vaginally.

Demonstration of the proper cleaning of the applicator was a key trial to minimize the risk of microbiological contamination to the patient and meeting hygiene requirements. Different easy-to-apply cleaning procedures in a home environment were evaluated for their effectiveness. Simulation of the application was performed by dipping the applicator with a placebo tablet into a newly developed artificial vaginal fluid. The microbiological status of the applicator was evaluated after the individual cleaning procedures and assessed against the requirements of the European Pharmacopeia for vaginal products.

The most effective cleaning procedure was applied in a multiple-use trial following the treatment scheme of applications with 24 simulated uses followed by cleaning after each use. The microbiological status after 24 simulated uses was still within the acceptance criteria of the European Pharmacopeia. By applying the developed cleaning procedure patient risk concerning microbiological contamination is minimized.

The mechanical stability trial included various batches of different age. Simulation of use was performed by placing a placebo tablet into the applicator and eject the tablets. This procedure was repeated up to 50 times per applicator. Predefined critical sections of the applicator that ensure proper functionality were visually assessed (microscope) after simulated multiple-use. Although examinations showed signs of wear, the applicators remained fully functional. The trial demonstrated that there is no patient risk concerning injuries due to multiple-use of an applicator.

All three trials performed produced results justifying and qualifying the applicator for the intended multiple-use. The procedures described might be a guide as to how this kind of medical device should be tested for suitability.

## Data Availability

Trial 1: All data and information are available at: HELM AG, Hamburg, Germany. Trial 2: Raw data of the cleaning verification study are available at: Labor LS SE & Co. KG, Bad Bocklet-Großenbrach, Germany. Trial 3: Raw data of the Mechanical Functionality and Stability Trial are available AT Manfred Schägner GmbH, Steinmauern, Germany.

## Abbreviations

CT: Clinical trial
LDPE: Low density polyethylene
PD: Pharmacodynamic
Ph. Eur.: European Pharmacopeia
PP: Polypropylene
TAMC: Total aerobic microbial count
TYMC: Total yeast and mold count
